# UHPose-VAD: Unsupervised Video Anomaly Detection via Pose-Graph Learning and Normalizing Flow

**DOI:** 10.3390/jimaging12060227

**Published:** 2026-05-27

**Authors:** Di Jiang, Huicheng Lai, Guxue Gao, Dan Ma, Liejun Wang

**Affiliations:** 1 College of Computer Science and Technology, Xinjiang University, Urumqi 830017, Chinawljxju@xju.edu.cn (L.W.); 2 Key Laboratory of Signal Detection and Processing, Xinjiang Uygur Autonomous Region, Urumqi 830017, China; 3 Joint Laboratory of SilkRoad Multilingual Cognitive Computing International Collaboration, Urumqi 830017, China; 4 School of Computer and Software Engineering, Huaiyin Institute of Technology, Huaian 223003, China

**Keywords:** video anomaly detection, unsupervised learning, pose graphs, spatiotemporal modeling, Gaussian mixture model

## Abstract

Unsupervised video anomaly detection (VAD) aims to identify unusual events by learning from unlabeled videos. However, many current methods overlook the fine-grained spatiotemporal dynamics of human poses, which are crucial for detecting localized anomalies like falls or assaults. Prevailing methods that rely on raw RGB frames are often susceptible to variations in lighting and background and struggle to capture the precise structural relationships of human bodies over time. To bridge this gap, we propose UHPose-VAD, a novel unsupervised framework that integrates human pose dynamics with normalizing flow within a graph-based probabilistic model to capture anomalies through spatiotemporal Gaussian distributions. Our framework first extracts human pose keypoints and normalizing flow features. These are then modeled by a graph convolutional network that adaptively learns the graph connectivity, effectively mapping the data to a latent space. This approach allows the model to explicitly reason about the spatiotemporal relationships between body joints, making it inherently more robust and interpretable for human-centric anomaly detection. Finally, a Gaussian Mixture Model fits the latent features of normal training data, learning the intrinsic manifold of regular motion patterns. Extensive experiments on ShanghaiTech and UBnormal datasets show that UHPose-VAD achieves state-of-the-art performance among unsupervised methods, with AUC scores of 86.1% and 69.4%, respectively.

## 1. Introduction

With the proliferation of intelligent surveillance systems, video anomaly detection (VAD) has become a cornerstone technology for enhancing public safety [1,2], optimizing traffic management [3], and combating financial fraud [4]. Unlike conventional computer vision tasks, VAD is inherently challenging due to the open-set nature of anomalies—abnormal events are rare, context-dependent, and semantically diverse [5]. For instance, running is normal in a park but anomalous in a hospital hallway.

Despite the remarkable progress of deep learning in video anomaly detection [6], most state-of-the-art approaches still rely on supervised or weakly supervised learning, which demands large-scale datasets with frame-level annotations [7,8,9]. This reliance hinders scalability, as annotating anomalies is both labor-intensive and inherently subjective. Reconstruction-based methods often struggle with the complexity of high-dimensional video data, while prediction-based techniques fail to capture fine-grained spatiotemporal anomalies, such as subtle aggressive gestures. More importantly, existing methods frequently overlook human pose dynamics—an essential cue for detecting localized anomalies that cannot be effectively modeled using raw pixels or optical flow alone [10].

Training an effective anomaly detector typically requires large-scale datasets with fine-grained, frame-level annotations—a process that is both time-consuming and resource-intensive. This limitation has motivated increasing interest in unsupervised VAD approaches, which can eliminate the need for manual labeling. Lara et al. [11] leverage a temporal convolutional network to model video temporal structures and propose a method to constrain the large search space in the multiple instance learning (MIL) framework. Building on MIL with ranking, Xu et al. [12] introduce a temporal enhancement network to learn motion-aware features. Doshi et al. [13] design an anomaly regression network combined with two novel loss functions to detect anomalies online. De et al. [14] propose a self-attentive integrated conditional random field to model short-range correlations. Wu et al. [15] develop a multi-scale continuous perception refinement network to enhance MIL-based detection. Feng et al. [16] present a multi-instance self-training framework that generates segment-level pseudo-labels by selectively sampling sparse video fragments. Although these unsupervised or weakly supervised methods have achieved promising results, they often remain sensitive to background clutter, lighting variations, and other irrelevant contextual factors—limiting their robustness in complex real-world scenarios.

To address these limitations, we propose UHPose-VAD, a novel unsupervised framework that leverages spatiotemporal graph learning on human poses for video anomaly detection. It is important to clarify the scope of “unsupervised” in our work. Following the common practice in video anomaly detection, we consider a method unsupervised if it does not utilize any video-level or frame-level abnormality labels during training. However, like most modern VAD approaches, our framework leverages a pre-trained pose estimator (trained on large-scale static image datasets like COCO) to obtain robust human keypoint representations. This pre-training provides a strong anatomical prior that is task-agnostic—it encodes general knowledge about human body structure, not information about what constitutes an “anomaly” in a specific surveillance scene. This distinction is crucial: our contribution lies in how we unsupervisedly learn the manifold of normal motion patterns from pose sequences, without ever seeing annotated anomalies.

Our approach is motivated by the premise that human skeleton sequences provide a robust, high-level representation that is invariant to appearance changes and explicitly encodes structural and dynamic information. The proposed framework begins with a pose extraction (PE) module that obtains human keypoints, which are tracked to form temporal pose graphs. A normalizing flow feature mapping (NFFM) module enriches these graphs with motion context. Subsequently, our core normalizing flow graph convolution (NFGC) module adaptively learns the graph connectivity to model the latent spatiotemporal distribution of normal poses. Finally, a Gaussian Estimation (GE) module fits this distribution, allowing anomalies to be detected as significant deviations.

Our main contributions are summarized as follows:

- We propose a fully unsupervised VAD framework that integrates adaptive graph learning with flow-based density estimation in a novel way. Unlike prior works that treat graph construction and anomaly scoring separately, our framework jointly learns spatiotemporal dependencies and the normal motion manifold through end-to-end optimization, without requiring any manual annotations.
- We propose a NFGC module that dynamically learns spatiotemporal dependencies among body joints. Unlike prior works using fixed skeletal graphs, our module fuses three complementary adjacency matrices—attention-based dynamic matrix, globally learnable matrix, and static anatomical prior—to capture both physical constraints and latent motion synergies (e.g., coordinated limb movements during running) that are critical for detecting subtle anomalies.
- By coupling graph-based feature extraction with a Gaussian Mixture Model via normalizing flows, our method replaces heuristic anomaly scores (e.g., reconstruction error) with a rigorous likelihood-based metric, directly measuring deviations from the learned normal motion manifold. This probabilistic formulation provides more interpretable and theoretically grounded anomaly detection.
- Extensive experiments on ShanghaiTech and UBnormal datasets demonstrate that UHPose-VAD achieves new state-of-the-art AUC scores of 86.1% and 69.4%, respectively. We also provide comprehensive ablation studies, robustness analysis under realistic corruptions, and model complexity analysis to validate the effectiveness and efficiency of our approach.

## 2. Related Work

### 2.1. Unsupervised Video Anomaly Detection

Unsupervised video anomaly detection aims to learn patterns of normal behavior from unlabeled data, treating deviations from these patterns as potential anomalies. Early methods primarily relied on handcrafted features (e.g., histogram of gradients) and statistical models (e.g., Gaussian mixture models) to represent normality [17]. With the advent of deep learning, reconstruction-based methods—such as those using autoencoders [18] or generative adversarial networks (GANs) [19]—have become dominant. These approaches typically estimate anomalies based on reconstruction errors, under the assumption that abnormal inputs cannot be accurately reconstructed. However, due to the high-dimensional nature of pixel space, subtle anomalies may not significantly degrade reconstruction quality, leading to missed detections. To address this limitation, recent works have incorporated motion cues (e.g., normalizing flow) [20] or memory-augmented modules [21] to improve the modeling of temporal consistency and reduce feature redundancy. Nonetheless, most existing approaches still overlook fine-grained human dynamics, which are crucial for detecting localized pose-related anomalies such as falls, stumbles, or aggressive gestures.

### 2.2. Pose-Based Anomaly Detection

Human pose offers a compact and semantically meaningful representation for understanding anomalous behaviors. Early pose-based approaches [22] utilized skeletal keypoints as input to recurrent neural networks for anomaly classification, but these methods typically required labeled data. Subsequent unsupervised extensions [23] adopted graph neural networks (GNNs) to model the spatiotemporal relationships among body joints, improving robustness to background noise and visual clutter. However, many existing methods treat pose sequences as fixed graph structures, failing to account for dynamic inter-joint dependencies that evolve over time. To address this limitation, recent work [10] introduced hierarchical pose graphs to capture multi-scale joint interactions, though the graph construction process still relies on handcrafted heuristics. In contrast, our proposed framework automatically learns graph connectivity and edge weights from both human poses and normalizing flow, enabling adaptive and data-driven spatiotemporal modeling that better captures the complexity of human motion dynamics in anomaly detection.

### 2.3. Spatiotemporal Graph Learning

Graph-based approaches have gained increasing attention in video anomaly detection due to their ability to model structured relationships across space and time. Temporal graph neural networks [24] aggregate frame-level features sequentially but often overlook spatial dependencies between entities. In contrast, spatiotemporal GNNs [25] jointly capture both spatial and temporal correlations, offering more holistic representations. For pose-based anomaly detection, graph convolutional networks typically use predefined adjacency matrices based on the physical structure of the human body. However, such fixed topologies may fail to capture latent motion dependencies that vary across different actions or contexts. To improve flexibility, recent works [26] have explored learnable graph structures using attention mechanisms to dynamically optimize graph connectivity—though primarily within supervised frameworks.

In this work, we extend the paradigm of learnable graph modeling to the unsupervised setting by integrating normalizing flow–guided graph convolution with Gaussian mixture–based density estimation. This combination enables our model to capture both local pose dynamics and global motion context, enhancing its sensitivity to subtle, context-dependent anomalies.

Despite recent progress, current unsupervised VAD methods still face three key limitations: (1) An over-reliance on pixel-level features, which are vulnerable to lighting changes and background clutter; (2) Rigid graph topologies that cannot adapt to diverse motion patterns; (3) Heuristic anomaly scoring strategies (e.g., reconstruction error) that may not align well with true semantic deviations.

UHPose-VAD addresses these challenges by combining pose-driven spatiotemporal graph learning with probabilistic anomaly scoring based on data likelihood estimation, offering a principled and adaptive approach to unsupervised video anomaly detection.

## 3. Methodology

**Figure 1:**
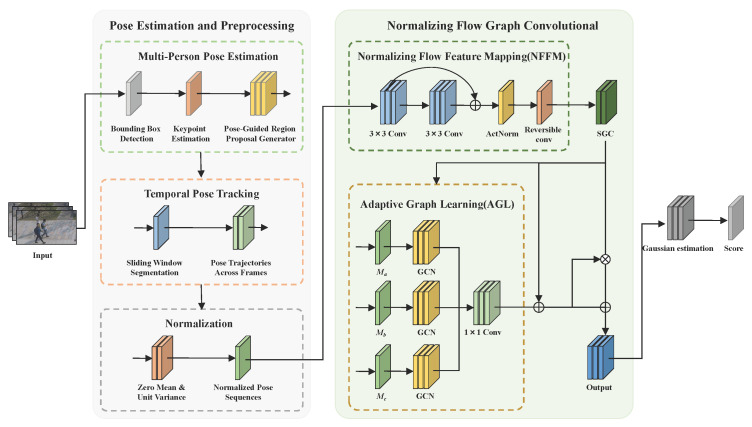
Architecture of UHPose-VAD: a pose-driven, graph-based framework for unsupervised video anomaly detection via Gaussian modeling.

We propose UHPose-VAD, an unsupervised framework for video anomaly detection that leverages human pose dynamics and spatiotemporal graph learning to model normal behavior patterns without requiring any annotated data. Specifically, our novelty lies in: (1) adaptively learning graph connectivity from pose sequences without any supervision, (2) coupling this with a flow-based invertible mapping to a latent space where a Gaussian mixture model captures multi-modal normal motion patterns, and (3) using a rigorous likelihood-based anomaly score instead of heuristic reconstruction errors. The framework is composed of four key components: pose extraction, normalizing flow feature mapping, normalizing flow graph convolution and Gaussian Estimation, as validated in Figure 1.

### 3.1. Pose Extraction

As illustrated in Figure 1, our approach operates under a fully unsupervised setting, where neither frame-level nor video-level anomaly labels are provided. To effectively model human motion patterns, we begin by extracting pose sequences from raw video inputs and constructing temporally aligned representations.

We first employ a region-based multi-person pose estimation framework on each frame of the video sequence *V*. Specifically, human instances are detected using bounding boxes, and their keypoint coordinates η are estimated via symmetric spatial transformation networks combined with parameterized pose non-maximum suppression to eliminate redundant or overlapping detections. To improve robustness and spatial consistency, we further utilize pose-guided region proposal generators that adaptively sample diverse pose configurations during training.

After detecting human poses in each segment $v_{i}$, we perform temporal tracking across consecutive frames to establish continuous pose trajectories throughout the video. A sliding window is applied to partition the video into overlapping clips of fixed length, ensuring consistent temporal granularity. Each pose sequence is then normalized to have zero mean and unit variance across joints and frames, which facilitates stable feature learning in subsequent modules.

To detect skeletons in each video frame independently, we utilize AlphaPose [27] with the YOLOX [28] detector. We then use PoseFlow [29] to track the skeletons across a video. After that, we divide each pose sequence into fixed-length segments using a sliding window approach. Finally, we normalize the pose coordinates by the image size to ensure scale invariance. This preprocessing stage enables the extraction of motion-relevant structural features while filtering out irrelevant pixel-level variations such as lighting and background clutter.

We acknowledge that AlphaPose, YOLOX, and PoseFlow are trained on large-scale labeled datasets (e.g., COCO for pose estimation). However, this does not violate the unsupervised setting of our framework for two reasons. First, these components are used only as fixed feature extractors; they are not fine-tuned on our target datasets, and they never access any anomaly labels (frame-level or video-level). Their role is to provide a generic human anatomical prior—knowledge about what a human skeleton looks like—which is task-agnostic and does not encode dataset-specific normality or abnormality. Second, the entire training process of UHPose-VAD operates without any anomaly annotations; we only use unlabeled video clips to learn the distribution of normal motion patterns. Therefore, while the pose estimator itself is pretrained in a supervised manner, our overall pipeline remains fully unsupervised for the task of anomaly detection. This practice is standard in the VAD literature and does not compromise the fairness of comparison with other unsupervised methods.

### 3.2. Normalizing Flow Graph Convolution

The normalizing flow graph convolution (NFGC) module processes the pose features extracted by the preceding pose extraction module. As illustrated in Figure 1, this process begins with normalizing flow feature mapping (NFFM), which transforms the input pose keypoints through a series of operations. Two successive 3×3 convolutional layers project the keypoints into a high-dimensional feature space, with the results fused back to the input via residual connections. An ActNorm layer is then applied to normalize the activations to zero mean and unit variance using trainable per-channel scale and offset parameters, thereby stabilizing training dynamics. Subsequently, a learned reversible 1×1 convolution, initialized as a random rotation matrix, permutes the channel order to enhance feature diversity while preserving information flow.

The features produced by the NFFM are then modeled by the core NFGC module. We construct an undirected spatiotemporal graph G=(H,L), where each node $H=\{H_{s,t}|s\in \left[1,\xi \right],t\in \left[1,\tau \right]\}$ represents a joint *s* at time step *t*, with ξ joints per frame and τ total frames. Each edge *L* models the spatial or temporal relationship between pairs of joints. The graph convolution operation is formulated as:

(1) $$f_{\mathrm{out}}\left(h_{s,t}\right)=\sum_{h_{s,t^{\prime }}\in \xi \left(h_{s,t}\right)}f_{\mathrm{in}}\left(h_{s,t^{\prime }}\right)\cdot \nu \left(h_{s,t},h_{s,t^{\prime }}\right),$$

where $\xi (h_{s,t})$ denotes the neighborhood of node $h_{s,t}$, and ν(·) represents the learned connection weights.

To comprehensively capture spatiotemporal relationships, NFGC leverages three complementary adjacency matrices. The attention-based adjacency matrix $M_{a}$ is a sample-specific, three-dimensional matrix computed using multiplicative attention. It is derived by embedding the input features twice using two sets of learned weights, followed by a transposed dot product and normalization to generate dynamic inter-joint dependencies. The global learnable adjacency matrix $M_{b}$ is a sample-invariant, fully learnable matrix updated over *K* iterations, capturing universal motion patterns consistent across all inputs. The static adjacency matrix $M_{c}$ is a fixed matrix that reflects prior human skeletal connectivity, shared across all layers. Each of these matrices drives a separate spatio-temporal GCN layer with independent weights. Their outputs are fused using a weighted summation:

(2) $$M=\alpha M_{a}+\beta M_{b}+\gamma M_{c}$$

where α,β,γ are trainable coefficients, thereby combining the strengths of dynamic patterns ($M_{a}$), global consistency ($M_{b}$), and anatomical priors ($M_{c}$).

The attention-based adjacency matrix $M_{a}\in R^{\tau \xi \times \tau \xi }$ captures sample-specific, dynamic inter-joint dependencies. It is computed using a multiplicative attention mechanism:

(3) $$M_{a}=\mathrm{softmax}\left(\frac{{\left(W_{Q}X\right)}^{T}\left(W_{K}X\right)}{\sqrt{d_{k}}}\right)$$

where $X\in R^{d\times \tau \xi }$ is the input feature matrix (with *d* denoting feature dimension), and $W_{Q},W_{K}\in R^{d_{k}\times d}$ are learnable weight matrices that project the input into query and key spaces. The scaling factor $\sqrt{d_{k}}$ stabilizes gradients during training. This formulation allows $M_{a}$ to dynamically assign high attention weights to joint pairs that exhibit strong motion correlation in the current sample, even if they are not directly connected in the human skeleton (e.g., left wrist and right ankle during walking). This adaptability is crucial for capturing diverse, context-dependent motion patterns.

Finally, the output features undergo a fusion and transformation step. They are split symmetrically along the channel dimension into two equal parts. One half is processed by an affine coupling layer (ACL), where it undergoes an invertible transformation for subsequent Jacobian matrix computation in probabilistic modeling. The other half is directly concatenated with features from an earlier stage, forming a residual connection that reinforces information flow across the network. This architectural design enables the NFGC to adaptively learn meaningful spatiotemporal structures from pose trajectories and effectively fuse them with normalizing flow–driven features, ultimately enhancing the model’s capacity to capture subtle and localized motion anomalies.

### 3.3. Gaussian Estimation

To probabilistically model the latent representations of human motions, we employ a Gaussian Mixture Model (GMM) framework. The core idea involves transforming the input data from the original space *X* to a structured latent space Z through an invertible mapping function *f* composed of the NFFM and NFGC modules. Specifically, a given pose sequence $\eta_{i}$ is mapped to a point $z_{i}=f\left(\eta_{i}\right)$ in Z, where we model the density as a mixture of Gaussian components to capture the multi-modal nature of normal human motions.

The transformation is governed by the change of variables formula. The probability density in the original data space *X* can be computed from the density in the latent space Z and the Jacobian determinant of the transformation *f*:

(4) $$p_{X}\left(v\right)=p_{Z}\left(f\left(v\right)\right)\cdot \left|\det\left(\frac{df}{dv}\right)\right|$$

For a composition of *K* invertible functions $f=f_{K}\circ f_{K-1}\circ \cdots \circ f_{1}$, the log probability decomposes additively, facilitating tractable computation:

(5) $$\log p_{X}\left(v\right)=\log p_{Z}\left(f\left(v\right)\right))+\sum_{i=1}^{K}\log\left|\det\left(\frac{df_{i}}{df_{i-1}}\right)\right|$$

In the latent space Z, we model the density as a Gaussian Mixture Model with *K* components:

(6) $$p_{Z}\left(z\right)=\sum_{k=1}^{K}\pi_{k}\;N\left(z;\mu_{k},I\right)$$

where $\pi_{k}$ are mixture weights satisfying $\sum_{k=1}^{K}\pi_{k}=1$, $\mu_{k}$ are component means, and *I* is the identity covariance matrix. The choice of identity covariance simplifies computation while maintaining sufficient flexibility to model complex distributions through the mixture, as the invertible transformation *f* is responsible for mapping the data to a space where Gaussian components with unit covariance are appropriate.

The GMM parameters $\pi_{k}$ (mixture weights) and $\mu_{k}$ (component means) are jointly optimized with the network parameters via the negative log-likelihood loss $L_{mle}$ (Equation (7)) using standard backpropagation. The identity covariance *I* is assumed for computational efficiency and to encourage the invertible transformation *f* to learn a disentangled latent representation where each component is isotropic. This design choice is common in normalizing flow literature and has been validated to be sufficient for density estimation when combined with a flexible flow [21].

The model is trained by minimizing the negative log-likelihood of the data:

(7) $$L_{mle}=-\log p_{Z}\left(f\left(v\right)\right))-\log\left|\det\left(\frac{df}{dv}\right)\right|$$

During inference, the anomaly score for a test instance is derived by computing $L_{mle}$ using Equation (7), which directly measures the deviation from the learned manifold of normal motion patterns. Instances that yield low likelihood under the GMM are classified as anomalies. This probabilistic formulation provides a principled and interpretable approach to anomaly detection, as the likelihood score has a clear statistical interpretation.

## 4. Experiments

To comprehensively evaluate the effectiveness of the proposed UHPose-VAD framework, we conducted extensive experiments on two publicly available benchmarks: ShanghaiTech [24] and UBnormal [30]. This section details our implementation setup, describes the characteristics of the evaluation datasets, and presents a comparative analysis against state-of-the-art methods.

### 4.1. Implementation Details

The experiments were conducted on an Ubuntu 22.04.4 operating system with an NVIDIA A40 GPU for model training and testing. All experiments were implemented using the PyTorch 2.1.2 framework. Input video frames were uniformly resized to 256×256 pixels and normalized to the range [−1,1].

For human pose extraction, we employ AlphaPose [27] with a YOLOX [28] detector pre-trained on COCO. The detector confidence threshold is set to 0.3, and non-maximum suppression (NMS) with an IoU threshold of 0.6 is applied to eliminate redundant detections. For temporal tracking across frames, we use PoseFlow [29] with the default parameters (tracking threshold = 0.2, matching window = 50 frames). When multiple people appear in a single frame, we select the person with the highest tracking confidence to maintain temporal consistency. However, if the tracking confidence of the currently selected person drops below a threshold (e.g., 0.5) and another person becomes more confident, we re-initialize tracking to the new person. This may cause identity switches across frames, but since we model each person independently and compute anomaly scores per frame, such switches do not negatively affect detection performance. For videos with no detected people, we use zero-padded pose features.

We partition each video into overlapping clips using a sliding window approach. Each clip contains τ=16 frames with a stride of 4 frames between consecutive clips. The pose coordinates are normalized by the image dimensions to the range [0,1], followed by zero-mean and unit-variance normalization across the training set.

The NFFM module consists of two 3×3 convolutional layers with 64 and 128 channels, respectively, followed by batch normalization and ReLU activation. The NFGC module employs 4 graph convolutional layers with 128 hidden units each. The GMM component number is set to K=3 based on validation performance. The GMM component means $\mu_{k}$ are initialized using k-means clustering on the latent features of the training set after 50 epochs of pre-training, then jointly optimized with the network via negative log-likelihood. The trainable fusion coefficients α,β,γ in Equation (2) are initialized to [0.4,0.3,0.3] and optimized jointly with the network parameters.

The network was optimized using the Adam optimizer with an initial learning rate of $1\times 10^{-4}$, weight decay of $5\times 10^{-4}$, and batch size of 32. The learning rate was reduced by a factor of 0.5 when the validation loss plateaued for 10 consecutive epochs. Training was conducted for 200 epochs, with early stopping applied if no improvement was observed for 30 epochs.

For evaluation, we adopted the widely used frame-level Area Under the Curve (AUC) of the Receiver Operating Characteristic (ROC) as our primary metric. Specifically, for each clip we compute the negative log-likelihood $L_{mle}$ (Equation (7)) as its anomaly score. For each frame *f*, the anomaly score $s_{f}$ is defined as the average of the scores of all clips that cover *f*:

(8) $$s_{f}=\frac{1}{\left|C(f)\right|}\sum_{c\in C(f)}L_{mle}^{\left(c\right)},$$

where C(f) is the set of clips containing frame *f*.

### 4.2. Datasets

We follow the official training and test splits for both datasets. For ShanghaiTech [24], the training set contains 274,515 normal frames from 13 scenes, and the test set contains 42,883 frames with both normal and anomalous events. For UBnormal [30], we use the standard split with 29 scenes for training (normal videos only) and 15 scenes for testing (containing both normal and abnormal events). No frame-level or video-level anomaly labels are used during training.

**Figure 2:**
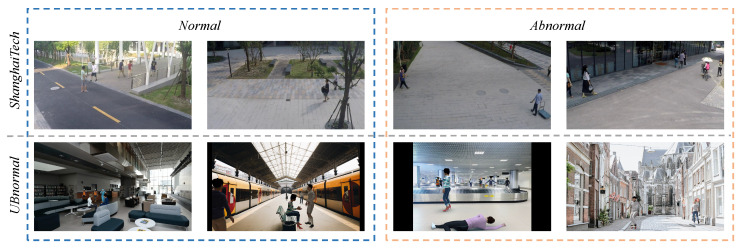
Example frames from the ShanghaiTech (**top**) and UBnormal (**bottom**) datasets. ShanghaiTech contains real-world surveillance videos with diverse camera viewpoints, while UBnormal provides synthetically generated anomalies with controlled motion patterns.

As shown in Figure 2, the ShanghaiTech dataset is a large-scale benchmark comprising real-world surveillance videos captured from various scenes on a university campus. It contains diverse normal activities (e.g., walking, waiting) and anomalous events (e.g., fighting, chasing, falling). Its challenging nature stems from complex backgrounds, varying lighting conditions, and diverse camera viewpoints, making it a standard and demanding testbed for evaluating video anomaly detection methods.

In contrast, the UBnormal dataset is a synthetically generated benchmark created using Cinema4D (Maxon Computer GmbH, Bad Homburg, Germany). It was designed to mitigate common issues in real-world datasets, such as annotation noise and severe class imbalance. UBnormal offers a controlled environment with a wide variety of structured abnormal events and diverse human motion patterns. It serves as a valuable benchmark for more rigorously assessing a model’s generalization capability and robustness to rare or unseen anomalies.

### 4.3. Comparison with State-of-the-Art Methods

We compare our proposed UHPose-VAD framework against a range of recent state-of-the-art methods on both datasets. The compared works span several years and represent a variety of technical approaches published in high-impact venues.

To ensure fair comparison, we follow the same evaluation protocol as prior works. All compared methods are evaluated using the official training/test splits of ShanghaiTech and UBnormal. Frame-level AUC is computed by comparing per-frame anomaly scores against ground truth frame-level annotations. For methods that report results on these benchmarks, we directly cite their published numbers. All results for our method are reported as mean ± standard deviation over 3 independent runs with different random seeds.

Table 1 presents a comprehensive comparison of our proposed UHPose-VAD against state-of-the-art methods on the challenging ShanghaiTech dataset. The results, organized chronologically from 2020 to 2025, reveal a clear trend of performance improvement over the years, with recent methods beginning to surpass the 80% AUC mark. Early approaches like MESDnet (73.2%) and scene (74.7%) established competitive baselines, while variational and dynamic modeling methods such as VABD (78.2%) and LLSH (77.6%) pushed performance further. The year 2023 witnessed remarkable breakthroughs: STG-NF [21] achieved 85.9% AUC, setting a new record, while SSMTL++ [31] also reached 82.9%. Subsequent methods such as Ristea et al. [32] in 2024 obtained 84.7% AUC, yet remained below the 2023 record. While other works have been proposed in the following years (such as in 2025), our proposed UHPose-VAD framework significantly outperforms all these predecessors, achieving a new state-of-the-art AUC of 86.1%. This consistent superiority over a wide range of methodologies—including generative models, self-supervised learning, and transformer-based architectures—strongly validates the effectiveness of our core design: explicitly modeling fine-grained human pose dynamics within a probabilistic framework to capture anomalies in complex, real-world surveillance scenes.

The performance comparison on the UBnormal dataset, as summarized in Table 2, further underscores the robustness and generalization capability of our method. UBnormal, with its synthetic yet highly diverse anomalies, presents a different set of challenges compared to ShanghaiTech. The performance trajectory here shows a notable jump from early MIL-based methods like MIL-Rank (54.1%) to more recent paradigms. Methods in 2021, such as RTFM [45], pushed the AUC to 66.8%, which remained a strong benchmark for subsequent years. Notably, prior to our work, the strongest results were achieved by STG-NF [21] with 71.8% AUC, followed by OCC-WS [46] (67.4%) and STPrompt [47] (63.9%). Our UHPose-VAD framework achieves a top AUC of 69.4%. This result is particularly significant as it demonstrates that our pose-centric and flow-integrated model generalizes effectively beyond real-world data to controlled synthetic environments. While this is 2.4% lower than STG-NF (71.8%) on UBnormal, we note that STG-NF uses a more complex normalizing flow architecture (which processes a larger feature dimension). On the real-world ShanghaiTech dataset, our method outperforms STG-NF (86.1% vs. 84.0%), suggesting that our design prioritizes robustness to pose noise over capacity for synthetic data. ﻿

### 4.4. Ablation Study

To quantitatively validate the contribution of each core component within the proposed UHPose-VAD framework, we conduct a series of ablation studies on both the ShanghaiTech and UBnormal datasets. The study is designed to isolate and evaluate the effects of the PE, NFFM, NFGC, and GE modules. The baseline performance is established by a simplified model, and components are incrementally added to assess their individual and collective impact on the final anomaly detection performance.

Table 3 shows the ablation study results conducted on the ShanghaiTech and UBnormal datasets to evaluate the contribution of each component in our proposed UHPose-VAD framework. The baseline model, which utilizes a simplified architecture without the specialized modules proposed in this work, yields AUC scores of 79.2% and 63.7% on the two datasets, respectively. The baseline model (first row in Table 3) consists of a simple 2-layer MLP with 256 hidden units and ReLU activation. It takes the concatenated pose keypoints (normalized coordinates of all 18 joints across 16 frames, flattened into a 576-dimensional vector) as input and outputs a reconstruction error (MSE between input and output) as anomaly score. No graph convolution, no GMM, and no normalizing flow is used. All other rows incrementally add the proposed modules (PE: pose extraction, NFFM: normalizing flow feature mapping, NFGC: normalizing flow graph convolution, GE: Gaussian estimation). This baseline achieves 79.2% AUC on ShanghaiTech and 63.7% on UBnormal, demonstrating that even a simple pose-based model is effective, but our full model significantly improves upon it. Notably, configurations that omit the PE module (rows 5–7) consistently underperform compared to their pose-based counterparts, highlighting the indispensability of explicit pose dynamics for human-centric anomaly detection. The full model, incorporating all four components (PE, NFFM, NFGC, GE), achieves the highest AUC scores of 86.1% on ShanghaiTech and 69.4% on UBnormal, demonstrating that each module contributes uniquely and that their synergistic integration is crucial for achieving state-of-the-art performance.

**Figure 3:**
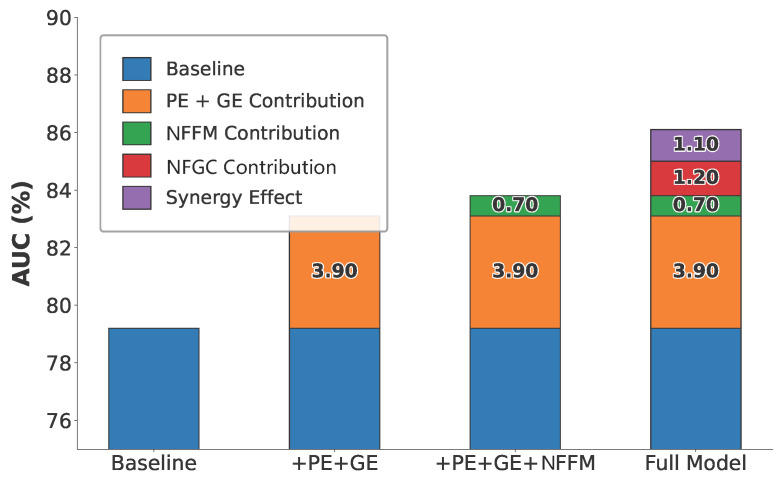
Module contribution research of PE, NFFM, NFGC and GE on the ShanghaiTech.

Figure 3 presents a stacked bar chart that visualizes the incremental contributions of each module within the proposed UHPose-VAD framework to the overall anomaly detection performance on the ShanghaiTech dataset, measured in AUC (%). The x-axis represents the progressive integration of modules, starting from a baseline without any modules (AUC = 79.2%), followed by PE, then GE, then NFFM, then NFGC. The y-axis indicates the cumulative AUC achieved by the model.

Each bar is composed of five stacked components: The baseline (blue) corresponds to a simple model that uses only pose extraction (PE) without graph modeling or GMM, achieving an AUC of 79.2%. This baseline does not include NFFM, NFGC, or GE modules. The subsequent bars add components incrementally. The PE + GE contribution (orange) shows that adding the GE module significantly improves performance by +3.9%, highlighting the importance of probabilistic modeling in capturing the normal motion manifold. The NFFM contribution (green) indicates that the addition of normalizing flow features contributes an additional +0.7%, underscoring the benefit of integrating fine-grained motion cues. The NFGC contribution (red) demonstrates that introducing NFGC further improves AUC by +1.2%, validating that adaptive graph-based mechanisms enhance the modeling of spatiotemporal relationships. Finally, the synergy effect (purple) reflects an additional +1.1% improvement, indicating that the joint optimization of all modules leads to performance gains beyond their simple additive contributions. Overall, the full model achieves a final AUC of 86.1%, demonstrating the effectiveness of each individual component as well as their collaborative integration.

### 4.5. Ablation Study on Graph Adjacency Matrices

To understand the contribution of each adjacency matrix in the NFGC module, we conduct a fine-grained ablation study where we individually remove or retain each matrix type. Table 4 reports the results on both datasets.

The results demonstrate that each matrix contributes uniquely to the overall performance. The full model achieves the highest AUC, confirming the complementary nature of the three adjacency matrices. Notably, removing $M_{a}$ (the attention-based dynamic matrix) causes a performance drop of 0.9% on ShanghaiTech, highlighting the importance of sample-specific connectivity for capturing diverse motion patterns. The global learnable matrix $M_{b}$ also plays a crucial role, with its removal leading to a 1.4% AUC decrease. Interestingly, the static anatomical prior $M_{c}$ provides a moderate contribution, and the model maintains reasonable performance even without it (−1.2%), suggesting that the learned matrices can partially compensate for anatomical priors while still benefiting from their regularization effect.

### 4.6. Qualitative Analysis

To provide intuitive insights into the temporal detection capability of our model, we present qualitative visualizations of anomaly scores on sequences containing anomalous events from both datasets. These curves demonstrate how UHPose-VAD responds to abnormal activities in the video stream and its precision in localizing such events temporally.

**Figure 4:**
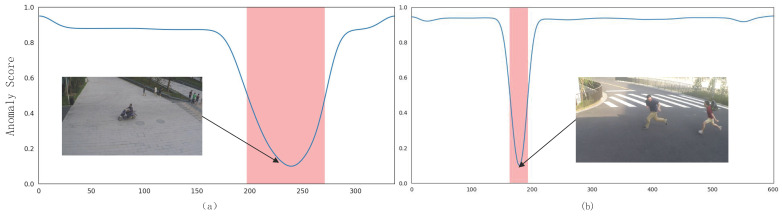
Anomaly score curves for two sequences from the ShanghaiTech dataset. (**a**) Biking; (**b**) Running. The blue curve represents the frame-wise anomaly scores predicted by UHPose-VAD, with higher values indicating greater abnormality. Red shaded areas mark the ground truth anomalous intervals. Both sequences contain real-world anomalous events that trigger significant peaks in the anomaly scores.

Figure 4 displays anomaly score curves for two representative sequences from the ShanghaiTech dataset. In both subfigures, the blue curve tracks the evolution of anomaly scores across frames, while red shaded regions indicate ground truth anomalous intervals. The results demonstrate UHPose-VAD’s effectiveness in real-world surveillance scenarios. In Sequence 1 (Figure 4a), the model produces a sharp, well-defined peak that closely aligns with the anomalous event, accurately capturing its onset and duration. Similarly, in Sequence 2 (Figure 4b), the anomaly score rises abruptly during the abnormal activity and quickly returns to baseline levels once normal behavior resumes. This pattern indicates the model’s sensitivity to deviations from learned normal patterns and its ability to temporally localize anomalies with precision.

**Figure 5:**
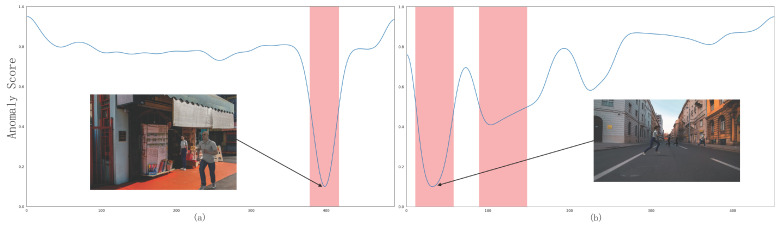
Anomaly score curves for two sequences from the UBnormal dataset. (**a**) Running; (**b**) Jaywalking (crossing the road). The visualization follows the same convention as Figure 4, with blue curves showing predicted anomaly scores and red areas indicating ground truth anomalies. The synthetic nature of UBnormal allows for controlled evaluation of specific anomaly types. The anomaly score per frame is computed as described in Section 4.1.

Figure 5 presents anomaly detection results on the UBnormal dataset, which contains synthetically generated but challenging anomalous events. In Sequence 1 (Figure 5a), the model demonstrates robust performance by maintaining low anomaly scores during normal periods while generating a prominent peak precisely aligned with the ground truth anomaly. Sequence 2 (Figure 5b) shows a more complex scenario where the anomaly score exhibits multiple peaks corresponding to different phases of abnormal activity yet still maintains strong temporal alignment with the ground truth intervals.

Across both datasets, several consistent patterns emerge. First, UHPose-VAD maintains low anomaly scores during normal activity periods, indicating effective learning of regular human motion patterns. Second, the model produces sharp, well-defined peaks during anomalous events, demonstrating precise temporal localization capabilities. Third, the magnitude of anomaly scores correlates well with the severity of behavioral deviations, with more dramatic anomalies triggering higher peaks. These qualitative observations align with our quantitative results and validate the effectiveness of our pose-centric approach in capturing semantically meaningful anomalies across diverse scenarios.

**Figure 6:**
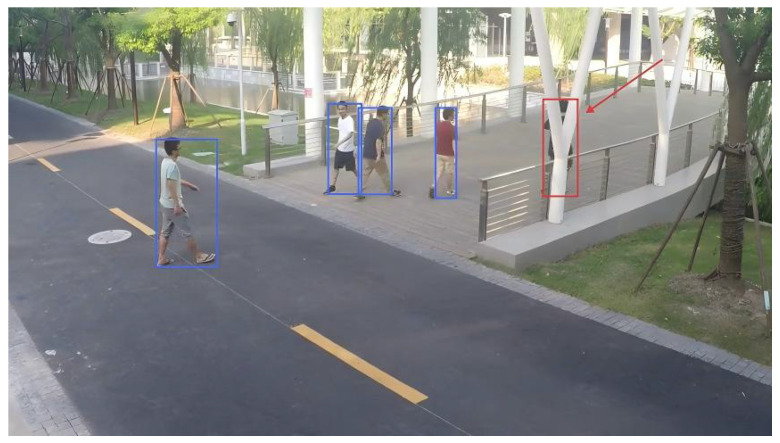
Failure case: severe occlusion leads to incomplete pose estimation and missed detection, Among them, blue represents normal detection, red represents unrecognized detection, and the red arrow points to the unrecognized detection. The anomaly score per frame is computed as described in Section 4.1.

Despite the strong overall performance, our method fails in certain scenarios. Figure 6 shows an example from ShanghaiTech where a person is severely occluded by a pillar. AlphaPose produces incomplete keypoints (missing left arm and leg), causing the pose graph to lose critical structural information. Consequently, the anomaly score remains low even though an abnormal event (a sudden fall) occurs. This highlights the dependency of our framework on accurate pose estimation. Future work could incorporate temporal smoothing or multi-frame keypoint interpolation to mitigate such failures.

### 4.7. Robustness Analysis

To evaluate the robustness of our proposed UHPose-VAD framework against potential data corruptions in real-world deployment scenarios, we systematically introduce and analyze three types of noise perturbations on the input data. Spatial noise is introduced by adding independent Gaussian noise with a standard deviation of 0.2 to the upper half of each video frame, simulating partial occlusion. Temporal noise is simulated via a random frame-dropping strategy with a discard rate of 0.1, emulating unstable frame rates or intermittent sensor failures. Furthermore, a combined spatio-temporal noise condition is created by applying both perturbations simultaneously, representing a more complex and challenging degradation scenario. The performance of our model under these adverse conditions is quantitatively assessed and reported in Table 5.

The robustness evaluation results in Table 5 demonstrate the remarkable resilience of UHPose-VAD to various data corruptions. On the ShanghaiTech dataset, the model maintains high performance under spatial noise (85.6% AUC), showing only a marginal decrease compared to the clean data performance (86.1%). This minimal degradation underscores the advantage of our pose-driven approach, which focuses on structural human dynamics rather than raw pixel appearances, making it inherently robust to localized visual corruptions like partial occlusions. Similarly, temporal noise causes negligible performance drop (84.7% AUC), indicating that the graph-based temporal modeling effectively compensates for intermittent missing frames. Most notably, even under the challenging combined spatio-temporal noise, our method achieves 84.2% AUC on ShanghaiTech and 66.9% on UBnormal, exhibiting excellent stability. The consistent performance pattern observed on both datasets confirms that our framework maintains strong anomaly detection capability even in non-ideal conditions, highlighting its practical suitability for real-world surveillance applications where data quality cannot be guaranteed.

### 4.8. Computational Efficiency

We evaluate the model complexity and inference speed of UHPose-VAD. Our method achieves a compact architecture with only 2.6M parameters, significantly lower than most existing approaches. Moreover, UHPose-VAD runs at 32 FPS on a single GPU (NVIDIA A40 GPU), demonstrating its suitability for real-time anomaly detection applications. This efficiency stems from the lightweight GCN-based backbone and the effective fusion mechanism that avoids heavy computation overhead.

As shown in Table 3, the model complexity increases moderately with each added component, and the full model remains lightweight (2.6M parameters) and real-time (32 FPS). The inference speed of all variants is sufficient for practical deployment.

### 4.9. Sensitivity Analysis

#### 4.9.1. GMM Components

The number of Gaussian components *K* in the GMM (Equation (6)) is a key hyperparameter that determines the model’s capacity to capture multi-modal normal motion patterns. We evaluate the sensitivity of UHPose-VAD to different *K* values on the ShanghaiTech validation set.

**Figure 7:**
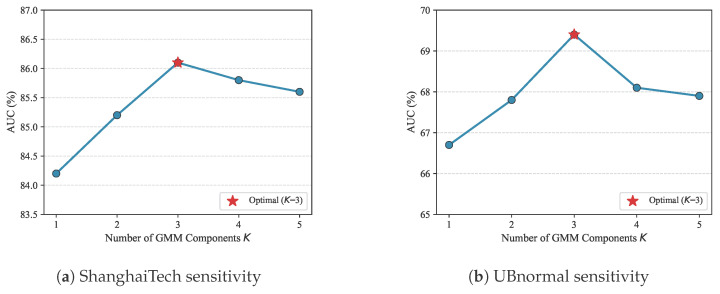
Performance variation with different numbers of GMM components *K* on ShanghaiTech (**a**) and UBnormal dataset (**b**). The optimal performance is achieved at K=3.

As shown in Figure 7, the model achieves its best performance with K=3 components. Using a single component (K=1) yields a lower AUC of 84.2%, confirming that normal human motions exhibit multi-modal characteristics that cannot be adequately captured by a single Gaussian distribution. Increasing *K* beyond 3 leads to a slight performance degradation (−0.3% at K=4, −0.5% at K=5), which can be attributed to overfitting to the training data. Based on this analysis, we set K=3 for all experiments.

We also evaluate the sensitivity of *K* on the UBnormal dataset. As shown in Figure 7b, the optimal *K* remains 3, achieving 69.4% AUC, while K=1 yields 66.7% and K=5 yields 67.9%. This confirms that the multi-modal nature of normal motion patterns is consistent across datasets.

#### 4.9.2. Graph Fusion Coefficients

To understand how the proposed UHPose-VAD balances the three adjacency matrices ($M_{a}$, $M_{b}$, $M_{c}$) and to verify whether the model truly favors adaptive connectivity over fixed physical constraints, we conduct a sensitivity analysis on the trainable coefficients α,β,γ in Equation (2). We fix two coefficients at their learned values while varying the third across the range [0,1], and observe the resulting AUC on the ShanghaiTech validation set.

**Figure 8:**
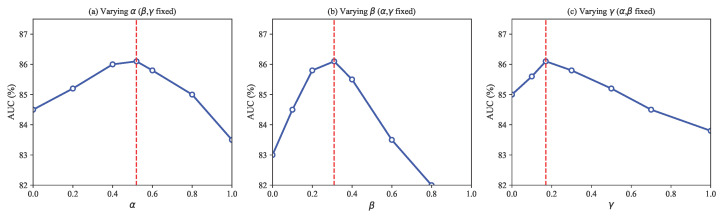
Sensitivity analysis of graph fusion coefficients on ShanghaiTech dataset. The plots show AUC variation when (**a**) varying α with β,γ fixed, (**b**) varying β with α,γ fixed, and (**c**) varying γ with α,β fixed. The red dashed lines indicate the final learned values.

As shown in Figure 8a, the model performance is relatively stable across different α values, with a broad plateau around the learned optimum (α≈0.52). This indicates that the attention-based dynamic connectivity consistently contributes meaningful information. In contrast, Figure 8b reveals that performance drops sharply when β deviates from its optimal range, highlighting the critical role of the global learnable matrix in capturing universal motion patterns. Interestingly, Figure 8c shows that the model maintains reasonable performance even when γ is reduced to zero, suggesting that while the anatomical prior $M_{c}$ provides useful regularization, the model can partially compensate for its absence through the learned matrices. Overall, the learned coefficients converge to a balanced configuration (α=0.52,β=0.31,γ=0.17), confirming that our adaptive fusion mechanism effectively combines the strengths of all three connectivity patterns.

## 5. Discussion

Our experimental results reveal several insights beyond the immediate performance comparisons. First, the sensitivity analysis on graph fusion coefficients (Figure 8) shows that performance drops sharply when β deviates from its optimum, while the model remains relatively robust to variations in α and γ. This suggests that the global learnable matrix $M_{b}$ plays a more critical role than the attention-based $M_{a}$ or the anatomical prior $M_{c}$ in capturing universal motion patterns. Interestingly, even when γ=0, the model maintains competitive AUC scores (85.2% on ShanghaiTech), indicating that the learned matrices can partially compensate for missing anatomical priors.

Second, the GMM sensitivity analysis (Figure 7a) confirms that normal human motions exhibit multi-modal characteristics, as K=3 significantly outperforms K=1. However, K>3 leads to overfitting, suggesting that the intrinsic modes of normal motion in surveillance scenarios are limited.

Third, the robustness analysis (Table 5) demonstrates that our pose-centric design is inherently resilient to spatial noise (e.g., partial occlusion), with only a 0.5% AUC drop on ShanghaiTech. This is a key advantage over pixel-based methods that would suffer more severely from such corruptions.

Nevertheless, the framework’s performance remains tied to pose estimation quality. In dense crowds or severe occlusions where AlphaPose fails, our method degrades gracefully but still suffers. Future work will explore (1) cross-frame temporal smoothing to filter erroneous pose detections and (2) extend the framework to model multi-person interactions via graph-based relational reasoning.

## 6. Conclusions

This paper presents UHPose-VAD, an unsupervised video anomaly detection framework that fuses human pose dynamics with normalizing flow features through a graph-based probabilistic model. The core contributions include: (1) a normalizing flow-guided graph convolution (NFGC) module that adaptively learns spatiotemporal dependencies via three complementary adjacency matrices, and (2) a Gaussian mixture model (GMM) in the latent space to capture multi-modal normal motion patterns. Extensive experiments on ShanghaiTech and UBnormal demonstrate that UHPose-VAD achieves state-of-the-art performance with AUC scores of 86.1% and 69.4%, respectively. Sensitivity analysis confirms that the global learnable matrix $M_{b}$ plays a critical role, while the model remains robust to spatial noise and missing anatomical priors. These results validate that explicit modeling of fine-grained pose dynamics offers a compelling alternative to pixel-centric approaches. Future work will extend the framework to multi-person interaction modeling and explore temporal smoothing to mitigate pose estimation errors in crowded scenes.

## Figures and Tables

**Table 1:** Comparison of Video Anomaly Detection in Terms of AUC Metrics Results on ShanghaiTech. “Unsup”: unsupervised (no labels); “Weak”: weakly supervised (video-level labels). Our method is fully unsupervised.

Year	Method	Journal/Conference	Supervision	AUC (%)
2020	MESDnet [33]	TMM	Weak	73.2
	Scene [34]	ACM MM	Unsup	74.7
2021	MONAD [13]	PR	Unsup	70.9
	VABD [35]	TIP	Unsup	78.2
2022	DLAN-AC [36]	ECCV	Weak	74.7
	LLSH [37]	TCSVT	Weak	77.6
2023	USTN-DSC [7]	CVPR	Weak	73.8
	SSAGAN [38]	TNNLS	Unsup	74.3
	FPDM [25]	ICCV	Weak	74.7
	SwinAnomaly [39]	Access	Weak	76.3
	SSMTL++ [31]	CVIU	Weak	82.9
	STG-NF [21]	ICCV	Unsup	85.9
2024	F2LM [40]	Access	Unsup	76.5
	Sun et al. [41]	PR	Unsup	73.1
	TSGAD [42]	WACV	Unsup	80.6
	Ristea et al. [32]	CVPR	Weak	84.7
2025	FusedVision [43]	CVPR	Weak	83.5
	Yang et al. [44]	PR	Weak	81.1
Ours	UHPose-VAD	-	Unsup	86.1 ± 0.2

**Table 2:** Comparison of Video Anomaly Detection in Terms of AUC Metrics Results on UBnormal. “Unsup”: unsupervised (no labels); “Weak”: weakly supervised (video-level labels). Our method is fully unsupervised.

Year	Method	Journal/Conference	Supervision	AUC (%)
2018	MIL-Rank [23]	CVPR	Weak	54.1
2020	AR-Net [48]	ICME	Weak	62.3
2021	BAF [49]	TPAMI	Unsup	59.3
	MIST [16]	CVPR	Weak	65.3
	RTFM [45]	ICCV	Weak	66.8
2023	DMU [50]	AAAI	Weak	59.91
	FPDM [25]	ICCV	Weak	62.7
	SSMTL++ [31]	CVIU	Unsup	62.1
	STG-NF [21]	ICCV	Unsup	71.8
2024	VadCLIP [51]	AAAI	Weak	62.3
	OPVAD [52]	CVPR	Weak	62.9
	STPrompt [47]	ACMM	Weak	63.9
	OCC-WS [46]	ECCV	Unsup	67.4
Ours	UHPose-VAD	-	Unsup	69.4 ± 0.3

**Table 3:** Ablation Studies Based on ShanghaiTech and UBnormal (✓: module used; -: not used).

PE	NFFM	NFGC	GE	AUC (%)	
				ShanghaiTech	UBnormal
-	-	-	-	79.2	63.7
✓	-	-	✓	83.1	67.9
✓	✓	-	✓	83.8	68.2
✓	-	✓	✓	83.6	67.8
-	✓	-	-	81.4	65.7
-	✓	✓	-	82.3	66.2
-	-	✓	-	80.7	65.1
✓	✓	✓	✓	86.1	69.4

**Table 4:** Ablation study on the three adjacency matrices ($M_{a}$, $M_{b}$, $M_{c}$) in the NFGC module.

Configuration	AUC (%)	
	ShanghaiTech	UBnormal
Only $M_{c}$	80.5	64.7
Only $M_{b}$	81.8	65.2
Only $M_{a}$	82.3	65.9
w/o $M_{c}$ (only $M_{a}+M_{b}$)	84.9	68.1
w/o $M_{b}$ (only $M_{a}+M_{c}$)	84.7	67.8
w/o $M_{a}$ (only $M_{b}+M_{c}$)	85.2	68.3
Full model ($M_{a}+M_{b}+M_{c}$)	86.1	69.4

**Table 5:** Model robustness evaluation under different noise conditions on ShanghaiTech and UBnormal datasets.

Scene	AUC (%)		
	Spatial Noise	Temporal Noise	Spatio-Temporal Noise
ShanghaiTech	85.6	84.7	84.2
UBnormal	68.0	67.1	66.9

## Data Availability

The datasets analysed during the current study, the ShanghaiTech dataset and the UBnormal dataset, are publicly available at their respective official repositories: https://svip-lab.github.io/dataset/campus_dataset.html (accessed on 21 May 2026) and https://github.com/lilygeorgescu/UBnormal (accessed on 21 May 2026).
